# Genomic insights into the alphaproteobacterium *Georhizobium* sp. MAB10 revealed a pathway of Mn(II) oxidation-coupled anoxygenic photoautotrophy: a novel understanding of the biotic process in deep-sea ferromanganese nodule formation

**DOI:** 10.1128/mbio.02675-24

**Published:** 2024-11-25

**Authors:** Xiuli Xu, Litao Zhang, Fuhang Song, Guoliang Zhang, Linlin Ma, Na Yang

**Affiliations:** 1 Key Laboratory of Polar Geology and Marine Mineral Resources (China University of Geosciences, Beijing), Ministry of Education; Hainan Institute of China University of Geosciences (Beijing); School of Ocean Sciences, China University of Geosciences, Beijing, P. R. China; 2 CAS Key Laboratory of Experimental Marine Biology, Institute of Oceanology, Chinese Academy of Sciences, Qingdao, P. R. China; 3 Key Laboratory of Geriatric Nutrition and Health, Ministry of Education of China, School of Light Industry Science and Engineering, Beijing Technology and Business University, Beijing, P. R. China; 4 Center of Deep Sea Research, Institute of Oceanology, Chinese Academy of Sciences, Qingdao, P. R. China; 5 Institute for Biomedicine and Glycomics, School of Environment and Science, Griffith University, Brisbane, Australia; McMaster University, Hamilton, Ontario, Canada

**Keywords:** *Georhizobium *sp. MAB10, Mn(II) oxidation-coupled anoxygenic photoautotrophy, photosynthetic reaction center II, multicopper oxidase, genome, deep-sea ferromanganese nodules

## Abstract

Under light conditions, Mn(II) facilitates the photoautotrophic growth of *Georhizobium* sp. MAB10, a strain derived from deep-sea ferromanganese nodules, along with the generation of dark Mn oxides (β-MnO_2_). This study investigated the genetic basis of Mn(II) oxidation-coupled anoxygenic photoautotrophy using genome sequencing and biochemical assays of strain MAB10. Preliminary results indicated the presence of genes encoding a functional pheophytin-quinone-type photosynthetic reaction center and a putative key enzyme for Mn(II) oxidation, namely FtsP/CotA-like multicopper oxidase GE001273. Under light conditions, Mn(II) significantly reduced the respiration rate and elevated the intracellular NADH/NAD_total_ ratio. This suggested that Mn(II)-derived electrons entered the cyclic photophosphorylation, partially replacing the oxidative phosphorylation for ATP production and enhancing the electron flow to complex I for NADH generation. *In vitro* enzymatic studies confirmed that GE001273 was a catalyst for Mn(II) oxidation in the outer membrane. Comprehensive genomic analyses of respiration and carbon and nitrogen metabolism revealed the high ecophysiological flexibility of strain MAB10 during Mn(II) oxidation-coupled anoxygenic photoautotrophy in deep-sea habitats. These analyses provided insights into bacterial Mn(II) oxidation-coupled anoxygenic photoautotrophy during microorganism-mediated deep-sea ferromanganese nodule formation.

**IMPORTANCE:**

Microorganisms are believed to participate in the biotic process of deep-sea ferromanganese nodule formation [Mn(II) oxidation]. Despite the multitude of studies and reviews focusing on the details of Mn(II) oxidation catalyzed by diverse heterotrophs, the mechanistic roles of manganese chemolithotrophs from ferromanganese nodules remain unclear. We demonstrate that strain *Georhizobium* sp. MAB10 can utilize Mn(II)-derived electrons for photoautotrophic growth, with concomitant generation of dark β-MnO_2_ type Mn oxides under near-infrared light condition. This study uses genomic and biochemical assays to explore the genetic basis of Mn(II) oxidation-coupled anoxygenic photoautotrophy. The comprehensive analyses of respiration and carbon and nitrogen metabolism further elucidated the high ecophysiological flexibility of strain MAB10 in deep-sea habits. These findings expand our understanding of the role of chemolithotrophs in deep-sea ferromanganese nodule formation and justify further investigations into the molecular basis for Mn(II) oxidation-coupled anoxygenic photoautotrophy.

## INTRODUCTION

Deep-sea ferromanganese nodules are increasingly being recognized as a potentially valuable economic resource, containing elements such as Fe, Mn, Co, Ni, Cu, Ti, and rare earth elements. They also serve as excellent materials for exploring biogeochemical cycling of these elements in abyssal seafloor environments (1). The formation mechanism of deep-sea ferromanganese nodules has remained elusive for 150 years since their discovery (2). Historically, emphasis has been placed on the abiotic processes (such as hydrogenetic and diagenetic processes); however, these abiogenic mechanisms alone do not fully explain the creation of ferromanganese nodules (3). With an increased understanding of microbial involvement in geochemical processes, research attention has shifted toward investigating whether biotic processes, particularly microbial metabolism, contribute to ferromanganese nodule formation (4–7). Mn-oxidizing bacteria, which oxidize Mn(II) to high-valent Mn oxides, appear to facilitate this process (3). Previous studies have extensively examined Mn(II) oxidation catalyzed by various heterotrophs, which can be enzyme-mediated or occur through chemical reactions with reactive oxygen species (e.g., superoxide or hydrogen peroxide) (8–11). In certain chemolithoautotrophic microorganisms, Mn(II) oxidation is coupled with aerobic energy conservation to meet energy requirements for growth (12). Recent evidence suggests that bacterial Mn(II) oxidation can also occur via a photosynthetic pathway under anoxic and light conditions (13, 14). Alphaproteobacteria isolated from diverse marine environments have demonstrated the ability to oxidize dissolved Mn(II) and produce particulate Mn oxides during heterotrophy (15). However, the physiological and metabolic roles of anoxygenic photoautotrophic alphaproteobacteria in deep-sea ferromanganese nodule formation remain poorly understood.

Bacterial anoxygenic photosynthesis utilizes light in longer visible and near-infrared ranges, with wavelength specificity varying among bacteria (16). These bacteria commonly employ organic carbon molecules for photoorganoheterotrophy, or utilize hydrogen, sulfide, elemental sulfur, or thiosulfate instead of water as photosynthetic electron donors for photoautotrophy (17). Metals such as As(III) and Fe(II) have also been identified as electron donors for anoxygenic photosynthesis via oxidization (18, 19). Among these, Mn(II)-related redox couples with redox potentials exceeding +500 mV are presumed to be the primary electron donors driving high potential phototrophic reactions to support bacterial growth; however, experimental confirmation is lacking (20).

Various forms of light such as bioluminescence, Cerenkov radiation, and thermal radiation have long been recognized at dark depths in the sea (21). In these regions, dense ferromanganese nodules have been identified in submarine mountain ranges and crustal fracture zones, where submarine lava eruptions are frequent, whereas they are notably absent from continental shelves and inland sea areas with rare submarine volcanoes (22). Thermal radiation from erupting superheated waters is the primary source of geothermal light in the deep sea (21, 23), particularly at wavelengths exceeding 700 nm. These wavelengths can be absorbed by the photosynthetic reaction centers of anoxygenic photoautotrophic bacteria and serve as the sole light source to support bacterial growth (24). Therefore, in addition to well-documented abiotic geological factors, it is anticipated that geothermal light-driven bacterial photosynthesis, coupled with Mn(II) oxidation, can play a role in catalyzing the formation of deep-sea ferromanganese nodules throughout Earth’s history.

In this study, we suggested a previously unrecognized contribution of bacterial anoxygenic photoautotrophy to the formation of deep-sea ferromanganese nodules. Through detailed sequencing and characterization of an alphaproteobacterial isolate from the genus *Georhizobium* (designated as MAB10), we elucidated the mechanisms underlying Mn(II) oxidation coupled with anoxygenic photoautotrophy. Moreover, our findings demonstrate the potential influence of photosynthetic processes on the global marine Mn(II) chemistry.

## MATERIALS AND METHODS

### Isolation, cultivation, and phylogenetic affiliation

Strain MAB10 was isolated from a deep-sea ferromanganese nodule sample (no. S4-TVG-3) collected by the Kexue vessel (NORC2020-581, 2020–2021) at the coordinates 138°44′07.0468″E, 14°12′21.6185″N. Immediately after collection, the ferromanganese nodule sample was immersed in a prepared aerobic basal medium, as outlined by Yu and Leadbetter (12), using ammonia (1 mM) and MnCO_3_ (20–50 mM) as nitrogen and Mn(II) sources, respectively. The sample was cultured for 6 months under light conditions at 28°C. After this enrichment period, 50 µL of the diluted culture was spread on basal medium plates supplemented with 18.0 g L^−1^ agar and incubated aerobically at 28°C for 3 months under light conditions. Individual colonies with distinct dark morphologies were picked using sterilized bamboo sticks and further cultured in a basal medium. Strain MAB10 was isolated and purified through multiple rounds of basal medium plating followed by 16S rRNA gene sequencing. To examine the anoxygenic photoautotrophy of strain MAB10, 50 mL of basal medium was cultured in a 150-mL flask at 28°C, shaken at 100 rpm, and exposed to a light intensity of 60–65 µmol m^−2^ s^−1^ under aerobic conditions. Growth was quantified by measuring the total protein concentration per mL using a bicinchoninic acid assay kit (cat. no. PC0020, Solarbio, China) (25). The 16S rRNA gene sequence of strain MAB10 was obtained following the method described by Song et al. (26). Phylogenetic trees were constructed using the neighbor-joining method with the MEGA software package (v10.0) (27).

### Genome sequencing, annotation, and comparison

Biomarker Technologies (Beijing, China) conducted genome sequencing using Oxford Nanopore Technologies for strain MAB10, yielding 634,526 sequences averaging 7,136 bp in length. Low-quality reads were filtered out using SMRT Link v5.0.1 software, and the obtained reads were assembled to generate one contig with an N50 size of 10,550 bp. Gene prediction was performed using Prodigal v2.6.3 (28). Gene functions were inferred from databases including Gene Ontology (29), Kyoto Encyclopedia of Genes and Genomes (30), Non-redundant Protein Database (31), Evolutionary Genealogy of Genes: Non-supervised Orthologous Groups (32), Pfam (33), Swiss-Prot, and TrEMBL (34).

Whole-genome comparisons between strain MAB10 and selected alphaproteobacteria strains were conducted by calculating average nucleotide identities (ANIs) and average amino acid identities (AAIs). The ANIs and AAIs were determined using the online ANI calculator tool and online AAI calculator tool (http://enve-omics.ce.gatech.edu/aai/index), respectively (35). The alphaproteobacteria selected for comparison, along with their GenBank accession numbers, are provided in the Supporting Information. The amino acid sequences of ribulose 1,5-bisphosphate carboxylase (RuBisCo) and their corresponding GenBank accession numbers for the neighbor-joining phylogenetic tree construction are included in the Supporting Information.

### Mn oxide examination

The strain MAB10 was aerobically cultivated in the basal medium supplemented with 30 mM MnCO_3_ at 28°C for 2 months. The morphological features of the strain and Mn oxides were assessed using a scanning electron microscopy-based energy dispersive system (SEM-EDS, S-3400N, Hitachi, Japan). Mn oxides were confirmed via a colorimetric leucoberbelin blue (LBB) assay following the method described by Zhang et al. (36). X-ray diffraction (XRD) analysis was conducted using an X-ray diffractometer (KYOWAGLAS-XA H-12, Rigaku, Japan) in the 2*θ* range of 10°–90°. The molecular changes in the Mn precipitates were identified using Fourier-transform infrared spectroscopy (FTIR, Nicolet IS50, Thermo Scientific, USA) within the wavelength range of 500–4,000 cm^−1^.

### Measurements of respiration and near-infrared light absorption

The respiratory capacity was assessed at 25°C using a Clark-type O_2_ electrode (Hansatech, Norfolk, UK), following the protocols outlined by Zhang and Liu (37). Briefly, a 2-mL sample was extracted directly from the culture system, shaken to equilibrate with atmospheric O_2_ levels, and placed into the assay chamber. Measurements were conducted with or without light over a 20-minute period. The near-infrared light absorption of photosynthetic reaction center II (RCII) at 820 nm was determined using an M-PEA instrument (Hansatech), with the modulated (33.3 kHz) near-infrared light provided by an optical density (OD_820_) light-emitting diode.

### Mn(II)-oxidizing activity assay

The *ge001273* (GenBank accession number: PP102380) from *Georhizobium* sp. MAB10 was amplified by PCR using the following primers: forward, 5′-CGCCCATGGATGTATATAGCGGAAAAC-3′ (with the underlined region indicating the NcoI site) and reverse, 5′-CCGCTCGAGGTTCTTCGGTATGAGATC-3′ (with the underlined region indicating the XhoI site). The PCR products were cloned into a pMD18-T vector (TaKaRa, Japan). After sequence confirmation, the PCR products were inserted into the multiple cloning site of the pET28a-sumo vector (Novagen, Germany), and the resulting plasmid was designated as pET28a-sumo::*ge001273*. Subsequently, the recombinant plasmid pET28a-sumo::*ge001273* was transformed into *Escherichia coli* strain BL21(DE3) cells to obtain the recombinant strain *E. coli* BL21(DE3)-pET28a-sumo-GE001273. The overexpressing strain *E. coli* BL21(DE3)-pET28a-sumo-GE001273 was cultured overnight and then inoculated into Luria-Bertani (LB) medium containing 50 µg mL^−1^ kanamycin at 37°C with shaking at 180 rpm. When the absorbance at 600 nm (OD_600_) reached approximately 0.5, the cells were induced with 0.5 mM isopropyl-D-thiogalactopyranoside and supplemented with 0.25 mM CuCl_2_. The temperature was changed to 16°C, and agitation was maintained for 20 h. *E. coli* cells overexpressing the recombinant GE001273 were harvested by centrifugation, washed, and resuspended in the HEPES buffer (10 mM, pH 7.5). After sonication and centrifugation at 12,000 × *g*, the supernatant was applied to a 5-mL HisTrap FF column (GE Healthcare, USA) equilibrated with the HEPES buffer (10 mM, pH 7.5). Elution with 300 mM imidazole yielded purified GE001273 at a final concentration of 1.38 mg mL^−1^. Recombinant GE001273 was assessed using sodium dodecyl sulfate-polyacrylamide gel electrophoresis on standard 10% gels, followed by Coomassie blue staining. The Mn(II)-oxidizing activity assay mixture comprised 0.55 µM of purified recombinant GE001273, 5 mM MnCl_2_, 0.25 mM CuCl_2_, and 10 mM HEPES (pH 7.5), and was incubated overnight at room temperature. Mn(II) oxidation activity was visualized by adding an LBB solution, and Mn oxides were quantified by measuring absorbance at 620 nm using a U–2900UV/VIS spectrometer (Hitachi, Japan) (38, 39). A standard curve was generated using known concentrations of KMnO_4_.

### Measurement of NADH/NAD_total_ ratio

MAB10 cells cultivated aerobically in basal medium, with or without 50 mM MnCO_3_, were collected by centrifugation. The intracellular NADH/NAD_total_ ratios (NAD_total_ = NAD^+^ + NADH) were determined using an NAD^+^/NADH assay kit with WST-8 following the manufacturer’s instructions (S0175, Beyotime Biotechnology, China). Briefly, cells were lysed with 400 µL of cold lysis buffer. To measure NADH levels, the lysed cell suspension was incubated at 60°C for 30 min to decompose NAD^+^ while preserving the NADH. Subsequently, 90 µL of alcohol dehydrogenase was added to 20 µL of untreated or heat-treated cell lysates and incubated at 37°C for 10 min. Finally, 10 µL of chromogenic WST-8 solution was added to each sample, and the mixture was incubated at 37°C for 30 min. Absorbance values were measured at 450 nm using a TECAN Infinite M1000 Pro multi-mode microplate reader (Switzerland) and compared with a standard curve generated simultaneously with the samples. The NADH/NAD_total_ ratios were calculated accordingly.

### Statistical analysis and data availability

All data were expressed as the mean ± standard error. The differences between experimental groups were assessed using one-way analysis of variance. The significance levels are indicated as follows: **P < 0.01, *P < 0.05.

## RESULTS AND DISCUSSION

### Genome of strain MAB10

**Fig 1:**
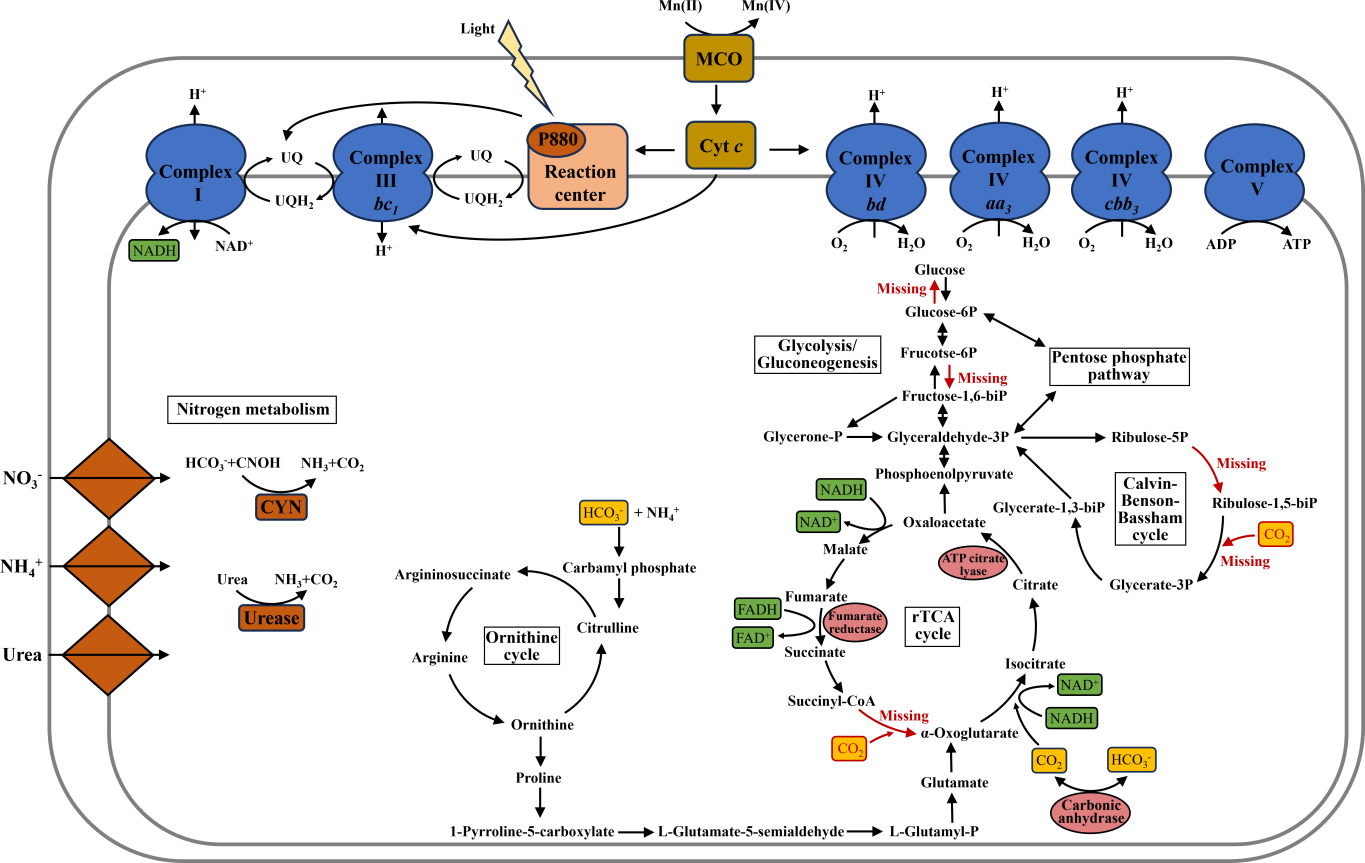
Schematic representation of Mn(II) oxidation-coupled anoxygenic phototrophy, respiration, and carbon and nitrogen metabolism in *Georhizobium* sp. MAB10. MCO, multicopper oxidase; Cyt *c*, cytochrome *c*; UQ, ubiquinone.

*Georhizobium* sp. MAB10, an alphaproteobacterium with short rod-shaped cells, was isolated from a deep-sea ferromanganese nodule by selecting photoautotrophs on a basal medium (Fig. S1). Its genome size was 4.04 Mb, containing 3,820 protein-coding genes with no evidence of a plasmid. Based on the 16S rRNA gene, strain MAB10 was most closely related to the reported isolates *G. profundi* WS11 and LR701459_s EC-SD404 (40), sharing 99.7% and 99.11% 16S rRNA gene sequence identity, respectively (Fig. S2). Whole-genome comparisons using ANI revealed that strain MAB10 shared 96.95% similarity with WS11, 88.62% similarity with LR701459_s EC-SD404, and <80% similarity with the other genomes investigated (Fig. S3A). The AAI comparisons presented a similar trend, with strains MAB10, WS11, and LR701459_s EC-SD404 exhibiting >90% identity with each other and <70% identity with other isolates (Fig. S3B). Genome annotation information for strain MAB10 is presented in Table S1. This study elucidated a complete set of genes associated with Mn(II) oxidation-coupled anoxygenic photoautotrophy, respiration, and carbon and nitrogen metabolism, which contribute to the high ecophysiological flexibility of *Georhizobium* sp. MAB10 during deep-sea ferromanganese nodule formation (Fig. 1; Table S2).

### Strain MAB10 formed manganese oxides during photoautotrophy

**Fig 2:**
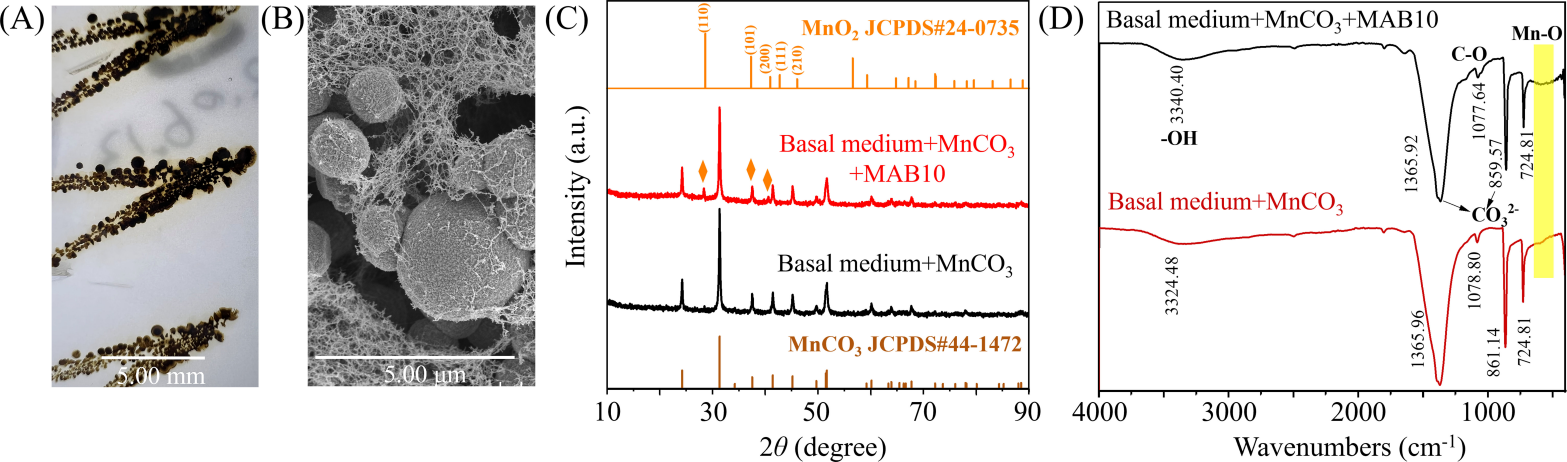
Analysis of Mn oxides formed by *Georhizobium* sp. MAB10. Culture plate image (**A**) and scanning electron microscope (SEM) image (**B**) of strain MAB10 in agarose-solidified basal medium supplemented with MnCO_3_. XRD pattern (**C**) and FTIR spectra (**D**) of Mn precipitates formed by strain MAB10 in liquid basal medium supplemented with MnCO_3_.

Several alphaproteobacteria have been documented for their ability to oxidize Mn(II) during heterotrophy (15). The genus *Georhizobium*, a relatively recent addition to the family Rhizobiaceae within the order Hyphomicrobiales, has only one described strain to date, *G. profundi* WS11, which was isolated from deep-sea sediment and is known to be heterotrophic (40). We observed that strain MAB10 exhibited the highest 16S rRNA gene similarity to *G. profundi* WS11. It thrived on a basal medium (utilizing ammonia and CO_2_ as the nitrogen and carbon sources, respectively) supplemented with MnCO_3_ under light, forming dark Mn oxides within 1 month (Fig. 2A). Mn(II) oxidation produced spherical particles of Mn oxides with hackly surfaces, ranging from 1 to 5 µm in diameter and displayed prominent Mn and O peaks (Fig. 2B; Fig. S4).

The specific composition of Mn precipitates during photoautotrophy of strain MAB10 was further examined via XRD, with the characteristic peaks depicted in Fig. 2C. The peaks at 2*θ* values of 24.25°, 31.34°, 51.64°, and 51.71° corresponded to the crystal form of MnCO_3_ (JCPDS#44-1472), consistent with the supplement in the basal medium (41). Moreover, the peaks at 28.37°, 37.53°, and 40.58° were attributed to MnO_2_ (JCPDS#24-0735), and the crystallographic analysis indicated a similarity to pyrolusite (β-MnO_2_) (42). This is in contrast to the reported Mn(II)-dependent autotrophic bacteria, which formed nodules similar to birnessite (12), indicating diverse Mn mineralization by autotrophic bacteria. The XPS results further validated the presence of Mn(VI), increasing from 15.8% to 24.28%, while Mn(II) decreased from 23.5% to 16.10% after inoculation with strain MAB10 (Fig. S5), confirming its role in β-MnO_2_ formation. Furthermore, molecular-level changes were investigated using FTIR, which revealed a new characteristic peak at 400–800 cm^−1^, corresponding to the Mn–O lattice vibration of MnO_2_ (Fig. 2D) (43). Previous studies have indicated the prevalence of pyrolusite in marine-derived ferromanganese nodules, similar to that in sample no. S4-TVG-3 used to isolate strain MAB10 in this study (Fig. S6) (44, 45). Combined with the ability of the strain MAB10 to form pyrolusite, this suggests its potential involvement in ferromanganese nodule formation.

### Photosynthetic reaction center II analysis

As strain MAB10 can produce β-MnO_2_ during photoautotrophic growth, it inherently possesses genes related to photosystems. Our analysis revealed that the genome of strain MAB10, similar to other phototrophic Proteobacteria, contained the RCII-encoding genes, including *puf*C, *puf*M, *puf*L, and *puh*A (46). Additionally, we detected one copy of the light- complex LH1. Strain MAB10 employed bacteriochlorophylls *a* and *b* for phototrophic growth, with the genes responsible for their biosynthesis dispersed throughout the genome (Fig. S7A). A complete pathway for biosynthesis of the major protective carotenoid, spirilloxanthin (normal and unusual spirilloxanthin), was also identified (Fig. S7B). Furthermore, the strain MAB10 contained several soluble mono-heme or di-heme cytochrome *c* family proteins, functioning as mobile electron carriers in photosynthetic pathways, such as cytochrome *c_553_* (11.60 kDa, 11.94 kDa, and 11.53 kDa) (47), cytochrome *c_556_* (15.68 kDa and 16.17 kDa) (48), and cytochrome *c_2_* (13.45 kDa) (Table S2) (49).

**Fig 3:**
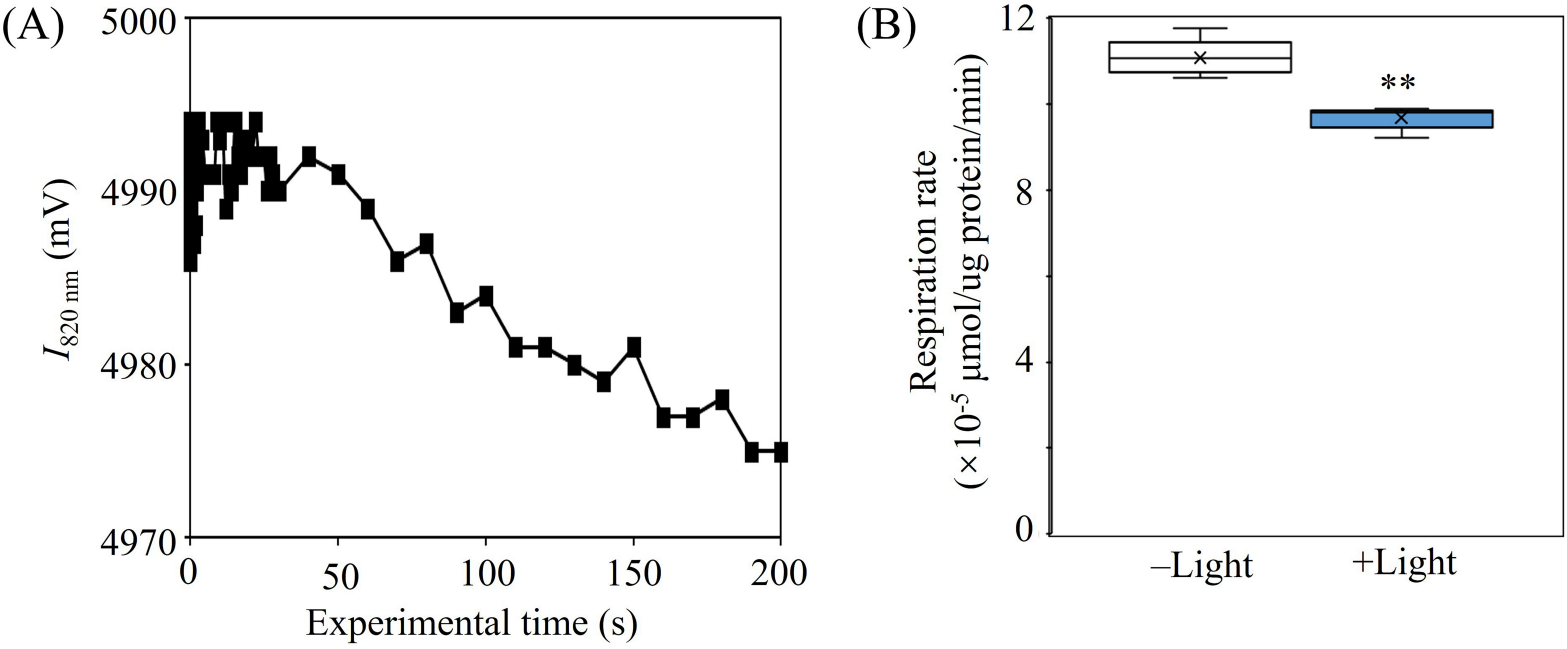
Role of RCII in *Georhizobium* sp. MAB10. (**A**) Near-infrared light absorption test of RCII. (**B**) Comparison of respiration rates of *Georhizobium* sp. MAB10 without Mn(II) under dark and light conditions (*n* = 5). **P < 0.01.

Additionally, we conducted experiments to verify the functionality of RCII during the photoautotrophic growth of strain MAB10 under light conditions. The near-infrared light absorption test demonstrated a decrease in light intensity, indicating the ability of RCII to absorb near-infrared light (Fig. 3A). Furthermore, the respiration rate notably decreased upon transferring the strain MAB10 from dark to light conditions (Fig. 3B), consistent with prior research suggesting that the additional ATP produced by RCII enables bacteria to lower their energy requirement from respiration (50). These findings confirm the role of RCII in supporting cell growth in the presence of near-infrared light.

### Mn(II) oxidation-coupled anoxygenic photoautotrophy

**Fig 4:**
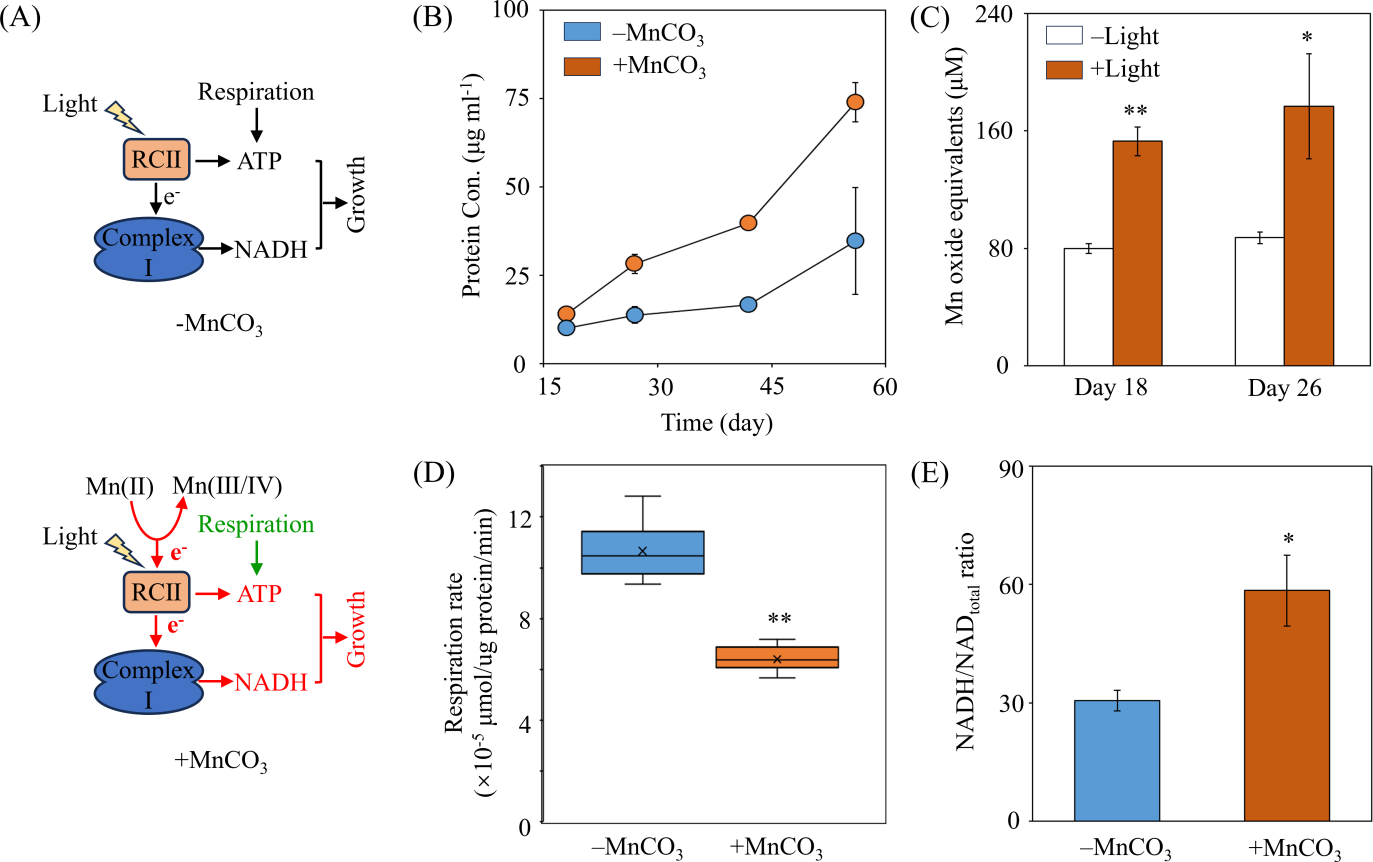
Mn(II) oxidation-coupled anoxygenic photoautotrophy of *Georhizobium* sp. MAB10. (**A**) Two diagrams illustrating the potential mechanisms of Mn(II)-derived electrons associated with ATP and NADH production via RC(II). Green lines and letters indicate downregulation. Red lines and letters indicate upregulation. (**B**) Growth of *Georhizobium* sp. MAB10 under light conditions (*n* = 3). (**C**) Mn(II) oxidation (*n* = 3). (**D**) Respiration on day 18 (*n* = 13). (**E**) NADH/NAD_total_ ratio (*n* = 3) on day 27. *P < 0.05, **P < 0.01.

Studies have revealed that oxygenic RCII, which can form manganese oxide particles under light, originates from anoxygenic RCII (51, 52). Additionally, Johnson et al. (14) demonstrated the presence of a transitional photosystem capable of the single-electron oxidation of Mn(II). A previous study highlighted the efficiency of low-potential Mn(II) as an electron donor in the native anoxygenic bacterium *Rhodobacter sphaeroides* (53). However, molecular understanding of Mn(II) as a photoautotrophic electron source and its coupling with anoxygenic photosynthesis to support bacterial growth remains limited. In our investigation of photoautotrophy in strain MAB10, we hypothesized that Mn(II) could be oxidized to Mn(III/IV) by Mn(II)-oxidizing proteins, followed by electron transfer to soluble cytochrome *c* family proteins to sustain cyclic photophosphorylation and bacterial growth (Fig. 1 and 4A). To validate this hypothesis, we initially assessed whether the biomass of strain MAB10 increased with the addition of Mn(II) under light conditions. Notably, we observed enhanced growth of strain MAB10 starting from the early exponential phase in basal medium supplemented with Mn(II) under light conditions (Fig. 4B). This growth stimulation by Mn(II) was observed in the Mn(II)-oxidizing alphaproteobacterium *Erythrobacter* sp. strain SD-21. However, different from MAB10, strain SD-21 exhibited only a slight growth increase, occurring only at the onset of the stationary phase (54). Additionally, we detected significantly increased Mn-oxide production under light conditions compared to dark conditions, implying a potential advantage of Mn(II) oxidation (Fig. 4C). One explanation could be the role of Mn(II) as a cofactor in various enzymes, aiding in cell protection against reactive oxygen species generated during photosynthesis (9, 55, 56). Therefore, Mn(II) may serve as a growth-limiting micronutrient under certain conditions. However, these explanations seem unlikely to account for the early exponential phase growth stimulation of MAB10 and the markedly increased Mn-oxide generation observed. An alternative explanation could be that Mn(II) can directly provide electrons to RCII via Mn(II) oxidation, thereby facilitating the growth of strain MAB10 (12). To test this hypothesis, we assessed the respiration rate of strain MAB10 following Mn(II) supplementation during photoautotrophic growth. Our observations revealed a significant reduction in the respiration rate of strain MAB10 under light conditions upon the addition of Mn(II) (Fig. 4D). Previous studies have indicated that anoxygenic photosynthetic bacteria can generate ATP via the cyclic photophosphorylation of RCII (20, 50). In addition, Mn(II) can serve as the primary electron donor for bacterial chemolithoautotrophy (12). The electrons derived from Mn(II) may participate in cyclic photophosphorylation, generating extra energy to enable strain MAB10 to partially substitute oxidative phosphorylation (respiration) with photophosphorylation, thereby reducing the respiration rate and enhancing growth efficiency. Moreover, we observed a notable increase in the intracellular NADH/NAD_total_ ratio of MAB10 cells from 30.58% to 58.46% with Mn(II) supplementation, indicating heightened electron flow to complex I for NADH generation (Fig. 1 and 4E). The elevated NADH levels subsequently entered the carbon fixation pathway, leading to increased biomass.

### Protein GE001273 is responsible for Mn(II) oxidation in the outer membrane

The strain MAB10 can utilize Mn(II)-derived electrons for growth and Mn-oxide formation, implying the presence of Mn(II)-oxidizing proteins encoded in its genome. Various bacteria capable of oxidizing Mn(II), whether heterotrophic or autotrophic, typically possess Mn(II)-oxidizing proteins such as multicopper oxidases, heme peroxidases, and cytochrome *c* type proteins (12, 57). Notably, the genome of strain MAB10 lacks homologs of outer membrane cytochrome *c* or porin-cytochrome *c* proteins, which are known to oxidize Mn(II) in obligate Mn(II)-dependent chemolithotrophic bacteria such as *Manganitrophus noduliformans* (12). This suggests that Mn(II) oxidation mechanisms in strain MAB10 may diverge significantly from those observed in reported obligate Mn(II) oxidation-dependent autotrophic strains.

**Fig 5:**
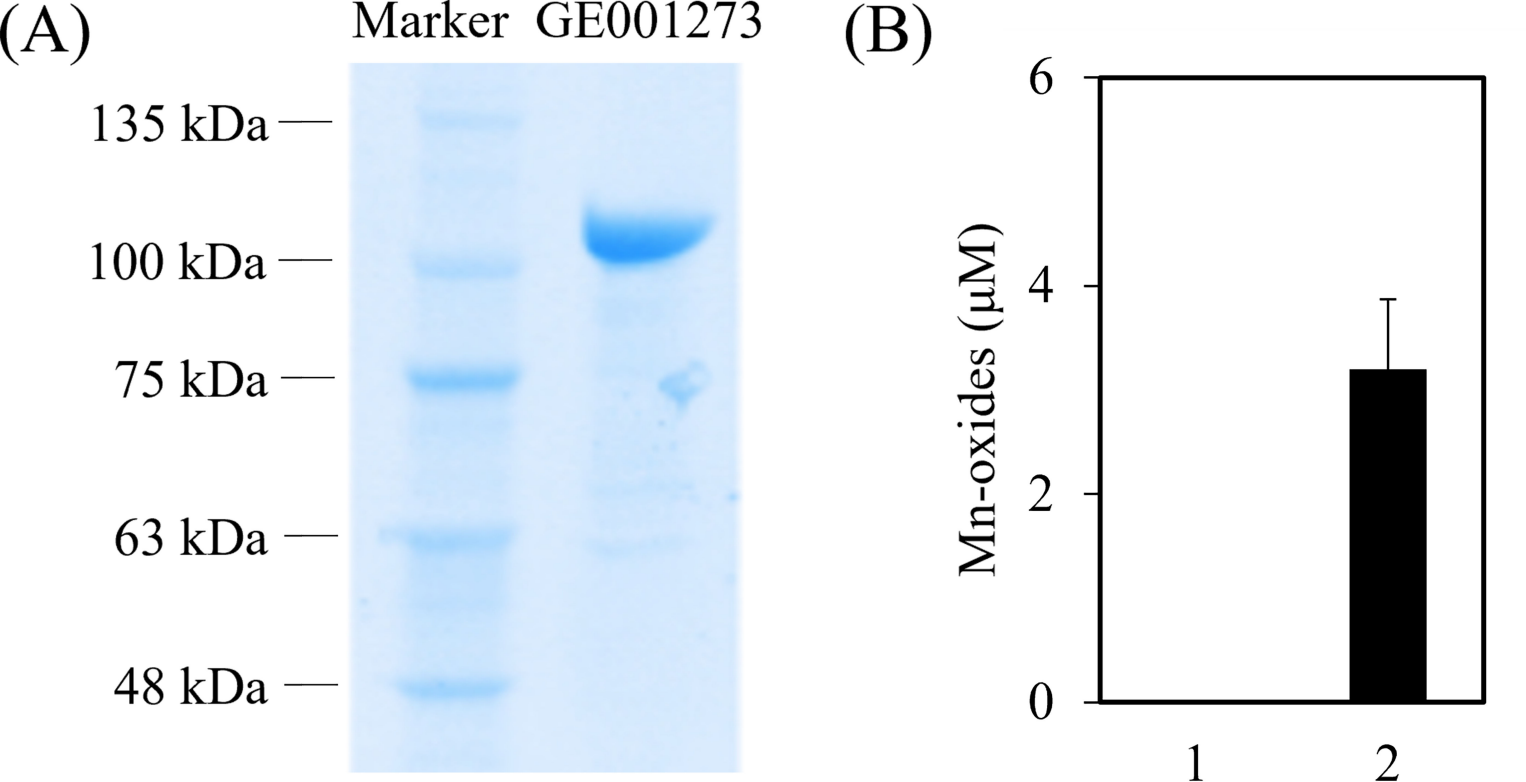
Purified recombinant GE001273 and Mn(II) oxidation activity assay. (**A**) Purified recombinant GE001273, as detected by SDS-PAGE and Coomassie blue staining. (**B**) GE001273 catalyzed the formation of Mn oxides (*n* = 4). 1, 10 mM HEPES buffer (pH 7.5) plus MnCl_2_ and CuCl_2_ reacting with LBB; 2, aliquots of 1 plus purified GE001273.

We found that strain MAB10 encoded a putative multicopper oxidase (GE001273, GenBank accession number: PP102380) comprising three cupredoxin domains (Fig. S8A) and four conserved Cu(II)-binding sites (Fig. S8B), similar to the structures of previously reported FtsP/CotA-like multicopper oxidase enzymes. This suggested that GE001273 functioned as a Cu-incorporated holoenzyme with potential Mn(II)-oxidation activity (57). Although GE001273 exhibited low identity (<50%) compared to previously reported FtsP/CotA-like Mn oxidases (Fig. S8B), it shared homologs with over 70% identities in bacteria such as *Rhizobium* spp., *Aurantimonas* spp., and *Ensifer* spp. These organisms can either oxidize Mn(II) directly or have a potential synergistic relationship with other species for Mn(II) oxidation. However, the detailed Mn(II) oxidation mechanism in these organisms remains elusive (15, 58, 59), making it unclear whether the FtsP/CotA-like multicopper oxidase plays a direct role in metal redox reactions. To address this uncertainty, we conducted an *in vitro* experiment to confirm the Mn(II) oxidation activity of protein GE001273. Recombinant GE001273 was expressed in *E. coli*, purified, and subjected to a Mn(II) oxidation activity assay to evaluate its catalytic function. Figure 5A illustrates the successful purification of recombinant GE001273 with a molecular weight of 100.7 kDa. The Mn(II) oxidation activity assay revealed a production rate of 5.84 ± 1.22 µmoL of Mn oxides per μmoL of GE001273 overnight (Fig. 5B; Fig. S9). These results confirm the direct involvement of GE001273 in Mn(II) oxidation in strain MAB10. Multicopper oxidases (such as GE001273) participate in extracellular electron transfer, complementing the role of cytochrome *c*-type proteins (60). Our analysis indicated that GE001273 contained three cupredoxin domains that are crucial for intermolecular electron transfer reactions (61). Additionally, based on CELLO predictions (62), GE001273 was anticipated to reside in the outer membrane, where it catalyzes Mn(II) oxidation and mediates the transfer of Mn(II)-derived electrons as a redox-active enzyme.

### Genes putatively involved in respiratory electron transport

After Mn(II) oxidation by GE001273, the resulting Mn(II)-derived electrons were probably channeled to RCII and respiratory complexes within the cytoplasmic membrane, facilitating the generation of NADH and ATP. This was supported by the notable increase in the intracellular NADH/NAD_total_ ratio observed in MAB10 cells (Fig. 4E). We identified genes associated with respiratory electron transport in the genome of strain MAB10. Specifically, a single gene copy encoding 14-subunit NADH-quinone oxidoreductase (complex I) was transcribed for heterotrophic respiration. Under photoautotrophic conditions, electrons originating from ubiquinol are likely redirected to respiratory complex I, thereby reducing NAD^+^ to NADH, which supports CO_2_ assimilation and biosynthesis (12). Strain MAB10 also possessed a complete ubiquinone biosynthesis pathway, consistent with the closest relative *G. profundi* WS11, where ubiquinone-10 served as the major quinone species involved in electron transport (40). Furthermore, strain MAB10 harbored one copy of the canonical cytochrome *bc_1_* complex (complex III), which facilitated electron transfer from ubiquinol to complex I for NAD^+^ reduction (63). Additionally, the presence of both high-affinity oxygen reductases (one copy of *cbb_3_*-type cytochrome *c* oxidase and one copy of cytochrome *bd* ubiquinol oxidase) and low-affinity oxygen reductases (two copies of *aa_3_*-type cytochrome *c* oxidase) (complex IV) allowed strain MAB10 to thrive in deep-sea environments characterized by fluctuating O_2_ levels during Mn(II) oxidation-coupled anoxygenic photoautotrophy. Detailed insights into these complexes and the F_1_F_0_-type ATP synthase (complex V) for ATP production are provided in Table S2.

### Genes putatively involved in carbon and nitrogen metabolism

**Fig 6:**
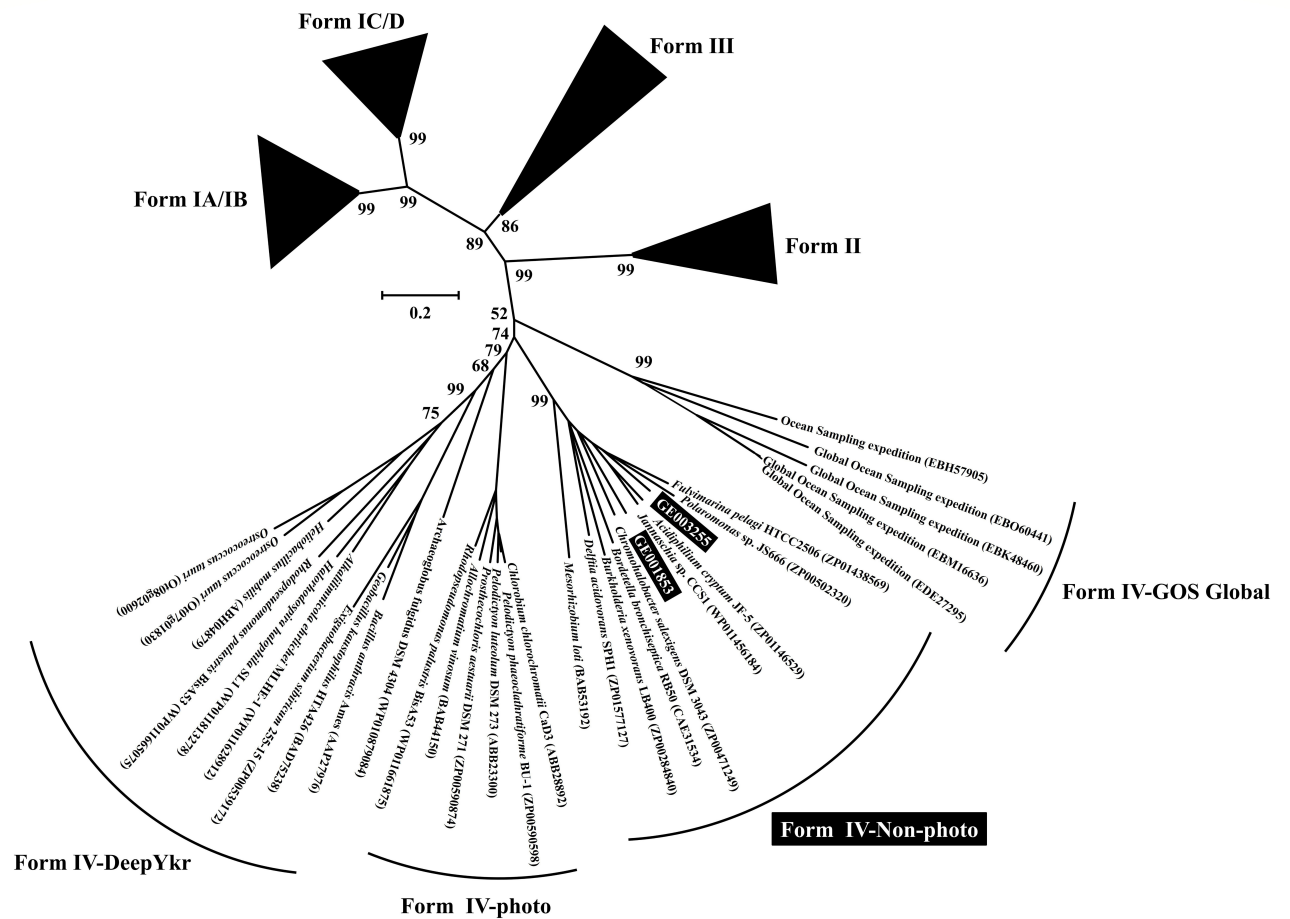
Neighbor-joining phylogenetic tree of RuBisCo form IV proteins based on the amino acid sequence of *Georhizobium* sp. MAB10 and other selected sequences. The sequences in this study are highlighted in black (GE003255 and GE001853), and the accession numbers in GenBank are shown in parentheses. Bootstrap values were calculated based on 1,000 replicates. The scale bar represents the number of base substitutions per site.

Having established that Mn(II) oxidation drives bacterial growth and metabolism via anoxygenic photoautotrophy, we investigated the genetic components responsible for the biomass production. Genome analysis revealed that strain MAB10 encoded genes associated with the Calvin-Benson-Bassham (CBB) cycle and the reductive tricarboxylic acid (rTCA) cycle for CO_2_ fixation (Table S2). Specifically, the genome contained two copies of genes (GE003255 and GE001853) encoding the RuBisCo large subunit. However, the key gene *prk*, which encodes phosphoribulokinase located immediately upstream of RuBisCo in the CBB cycle, was absent. Phylogenetic analyses based on the amino acid sequences indicated that these genes corresponded to the non-photo form IV RuBisCo (Rubisco-like protein) (Fig. 6). Consequently, putative RuBisCo in strain MAB10 may not be involved in the CBB cycle. Notably, forms II and III RuBisCos, unrelated to the CBB cycle, have been identified in archaeal genomes lacking the *prk* gene, where they function in nucleoside salvage or the reductive hexulose-phosphate pathway (64, 65). The detailed function of form IV RuBisCo in enabling strain MAB10 to sustain its Mn(II)-oxidation photoautotrophic lifestyle may require further investigation.

We observed that strain MAB10 lacked the crucial enzyme α-ketoglutarate synthase, which is essential for the complete operation of the rTCA cycle. Therefore, alternative mechanisms are likely to be used for CO_2_ fixation. In this study, NH_4_^+^ served as the sole nitrogen source for photoautotrophic activity of strain MAB10. Previous studies have suggested that NH_4_^+^ can facilitate the production of α-ketoglutarate via the ornithine cycle, thereby compensating for the absence of α-ketoglutarate synthase (66). Our genomic analysis revealed the presence of all genes necessary for α-ketoglutarate biosynthesis via the ornithine cycle in strain MAB10 (Fig. 1). Additionally, the genome of strain MAB10 contained a gene encoding carbonic anhydrase that facilitates the conversion of CO_2_ to bicarbonate and prepares it for cellular uptake.

Genomic analysis of strain MAB10 indicated the presence of all genes related to glycolysis/gluconeogenesis, except for phosphofructokinase and glucose-6-phosphatase. Consequently, strain MAB10 lacked the ability to utilize or produce glucose. Although bacteria typically prioritize glucose utilization because of their ability to facilitate rapid growth, the genome of strain MAB10 suggests alternative carbon source preferences (67). In addition, the genome of strain MAB10 lacked genes associated with glucose transport systems. Studies have shown that glucose is among the least favorable carbon sources for bacteria under nitrogen-poor conditions (68). The fluctuating nitrogen levels in marine environments may prompt strain MAB10 to favor alternative carbon sources to glucose. Notably, the genome contained a complete pentose phosphate pathway that facilitates NADPH production and provides intermediates for nucleotide biosynthesis.

Having demonstrated the ability of strain MAB10 to utilize NH_4_^+^ as the sole nitrogen source for growth during photoautotrophy (Fig. 4A), we identified an ammonium transporter in the genome. Through genome analysis, we discovered that strain MAB10 encoded the nitrate ABC transporter-related proteins and nitrogen regulatory two-component systems, NtrB-NtrC and NtrY-NtrX, facilitating the nitrate transport from extracellular to intracellular (69). However, the absence of enzymes responsible for nitrate or nitrite reduction, such as canonical nitrate or nitrite reductase, indicated the inability of strain MAB10 to utilize nitrate or nitrite (20, 70), which is consistent with the experimental findings. Notably, the biomass of strain MAB10 did not increase when the nitrate or nitrite was the only nitrogen source during photoautotrophy. Furthermore, the presence of genes encoding urease, cyanate hydratase, and the nitrilase suggests that a wide range of nitrogen sources were available to strain MAB10 (Table S2).

### Conclusion

**Fig 7:**
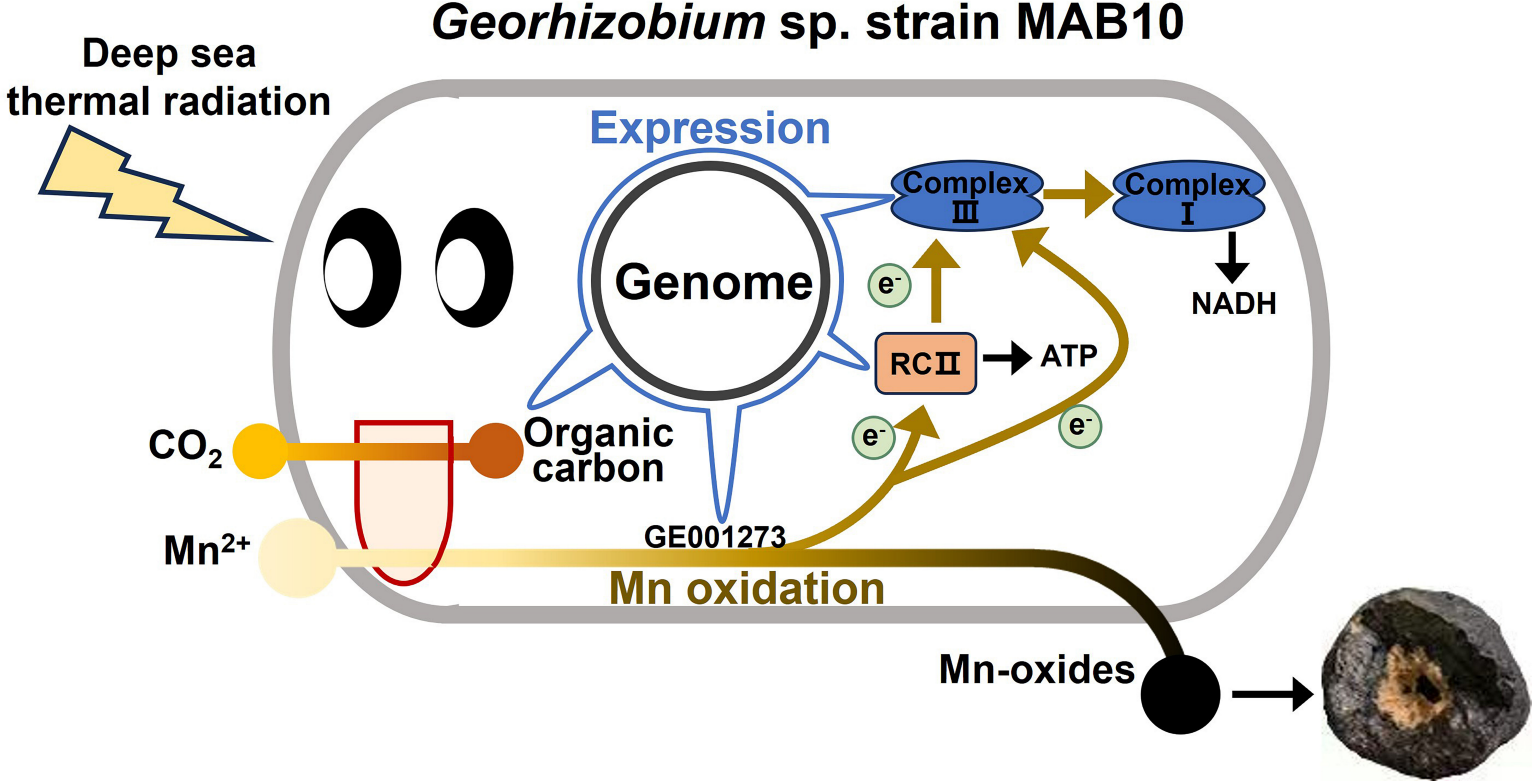
Schematic representation of Mn(II) oxidation-coupled anoxygenic photoautotrophy by *Georhizobium* sp. MAB10 cells.

In conclusion, genomic analysis of alphaproteobacterium *Georhizobium* sp. MAB10 has greatly expanded our understanding of Mn(II) oxidation-coupled anoxygenic photoautotrophy during the formation of deep-sea ferromanganese nodules. Strain MAB10 contains functional pheophytin-quinone-type photosynthetic reaction center RCII and FtsP/CotA-like multicopper oxidase GE001273, which are responsible for near-infrared light absorption and Mn(II) oxidation, respectively. This study confirms that Mn(II) oxidation can act as an energy-acquiring process coupled with RCII to produce NADH and ATP, supporting a photoautotrophic lifestyle and generating dark Mn oxides (Fig. 7). In addition, the genome reveals evidence of strain MAB10’s adaptation to deep-sea environments, including respiration and carbon and nitrogen metabolism. This study provides new insights into the formation mechanisms of deep-sea ferromanganese nodules from the perspective of biotic processes, particularly driven by Mn(II)-oxidizing autotrophs.

## Data Availability

The complete genome of *Georhizobium* sp. MAB10 has been deposited in GenBank under BioProject PRJNA934963 (accession number CP118314). The 16S rRNA gene sequence has been deposited in GenBank under accession number OR922347.

## SUPPLEMENTAL MATERIAL

Supplemental material mbio.02675-24-s0001.docx Table S2 and Figures S1–S9.

Table S1 mbio.02675-24-s0002.xls Genome annotation of strain *Georhizobium* sp. MAB10.

ASM does not own the copyrights to Supplemental Material that may be linked to, or accessed through, an article. The authors have granted ASM a non-exclusive, world-wide license to publish the Supplemental Material files. Please contact the corresponding author directly for reuse.
