# Supplementary material for: Genomic insights into the alphaproteobacterium Georhizobium sp. MAB10 revealed a pathway of Mn(II) oxidation-coupled anoxygenic photoautotrophy: a novel understanding of the biotic process in deep-sea ferromanganese nodule formation
Source: mBio. 2024 Nov 25;16(1):e02675-24. doi: 10.1128/mbio.02675-24 (PMC11708043; doi:10.1128/mbio.02675-24)
Supplement: Supplemental material — Table S2 and Figures S1–S9. [file mbio.02675-24-s0001.docx]

**Supplemental material**

**Genomic insights into an alphaproteobacterium** ***Georhizobium* sp. MAB10 revealed a pathway of Mn(Ⅱ) oxidation-coupled anoxygenic photoautotrophy: A novel understanding of biotic process in deep-sea ferromanganese nodule formation**

Xiuli Xu^1, #^, Litao Zhang^2, #^, Fuhang Song^3^, Guoliang Zhang^4^, Linlin Ma^5^, Na Yang^2, *^

^1^ Key Laboratory of Polar Geology and Marine Mineral Resources (China University of Geosciences, Beijing), Ministry of Education of China; School of Ocean Sciences, China University of Geosciences, 29 Xueyuan Road, Beijing, 100083, P. R. China

^2^ CAS Key Laboratory of Experimental Marine Biology, Institute of Oceanology, Chinese Academy of Sciences, 88 Haijun Road, Qingdao, 266400, P. R. China

^3^ Key Laboratory of Geriatric Nutrition and Health, Ministry of Education of China; School of Light Industry Science and Engineering, Beijing Technology and Business University, 33 Fucheng Road, Beijing, 100048, P. R. China

^4^ Center of Deep Sea Research, Institute of Oceanology, Chinese Academy of Sciences, 88 Haijun Road, Qingdao, 266400, P. R. China

^5^ Institute for Biomedicine and Glycomics, School of Environment and Science, Griffith University, Brisbane, 4111, Australia

^*^ Corresponding author:

Na Yang. CAS Key Laboratory of Experimental Marine Biology, Center for Ocean Mega-Science, Institute of Oceanology, Chinese Academy of Sciences, 88 Haijun Road, Qingdao, 266400, P. R. China. Telephone number: 8653282898562; E-mail address: [yangna@qdio.ac.cn](mailto:yangna@qdio.ac.cn).

^#^ These authors have contributed equally to this work and share first authorship.

**Supplemental Legends**

Page 3–5. Supplemental Methods used in this study.

Table S1 Genome annotation of strain *Georhizobium* sp. MAB10.

Page 6–7. Table S2 Identification of genes associated with pathways indicated in Figure 1.

Page 8. Figure S1 Transmission electron microscope image of strain MAB10.

Page 9. Figure S2 Neighbor-joining phylogenetic tree of full-length 16S rRNA genes of strains MAB10, WS11, and selected alpha-proteobacteria isolates.

Page 10. Figure S3 Heatmaps based on ANI and average AAI between strain MAB10 and selected alpha-proteobacteria isolates.

Page 11. Figure S4 Scanning electron microscope-energy dispersive system image of Mn oxides produced by strain MAB10.

Page 12. Figure S5 Mn (2p) spectrograms of Mn-precipitates with or without inoculation of strain MAB10.

Page 13. Figure S6 XRD analysis result of the deep-sea ferromanganese nodule sample (S4-TVG-3).

Page 14. Figure S7 Complete biosynthesis pathways of bacteriochlorophyll *a* and *b* and major carotenoid spirilloxanthin in strain MAB10.

Page 15. Figure S8 Amino acid sequence analysis of GE001273.

Page 16. Figure S9 GE001273 catalyzed the formation of Mn-oxides *in vitro*.

**Supplemental Methods**

**Morphology of strain MAB10.** The morphological feature of strain MAB10 were observed by a transmission electron microscopy (HT7800, Hitachi, Japan).

**Selected alphaproteobacteria** **and their GenBank accession numbers for whole genome comparison.** *Georhizobium profundi* WS11 (CP032509), LR701459 s EC-SD404 (LR701459), *Rhizobium subbaraonis* JC85 (OBQD01000085), *R. rhizolycopersici* DBTS2 (JABXYK010000001), *R. azooxidifex* Po 20/26 (JACIEE010000001), *R. alarense* TRM95111 (JAKGBU010000001), *Allorhizobium borborid* DN316 (JACIDU010000001), *R. rosettiformans* W3 (STGU01000001), *Shinella yambaruensis* MS4 (JALIRQ010000001), *R. halophytocola* YC6881 (JAGGJU010000001), and *Ensifer morelensis* Lc04 (CP015880).

**X-ray photoelectron spectroscopy**

The valence of Mn was determined using XPS (ESCALAB Xi+, Thermo Fisher Scientific, USA).

**Selected amino-acid sequences of RuBisCo and their GenBank accession numbers for neighbor-joining phylogenetic tree construction.** *Acidithiobacillus ferrooxidans* (AAD30508), *Alkalilimnicola ehrlichii* MLHE-1 (YP_743666), *Allochromatium vinosum* (P22859), *Hydrogenophilus thermoluteolus* (Q51856), *Hydrogenovibrio marinus* (Q59460), *Methylococcus capsulatus* Bath (AAU91176), *Nitrobacter vulgaris* (Q59613), *Nitrosomonas europaea* ATCC19718 (CAD85832), *Prochlorococcus marinus* CCMP1375 (AAP99596), *Rhodobacter capsulatus* (O32740), *Anabaena variabilis* ATCC29413 (ABA23512), *Chlamydomonas reinhardtii* (P00877), *Chlorella ellipsoidea* (BAA01765), *Crocosphaera watsonii* WH8501 (ZP_00516814), *Euglena gracilis* (NP_041936), *Gloeobacter violaceus* PCC7421 (BAC90097), *Nicotiana tabacum* (P00876), *Nostoc punctiforme* PCC73102 (ZP_00108159), *Spinacia oleracea* (P00875), *Synechococcus elongatus* PCC6301 (P00880), *Paracoccus denitrificans* PD1222 (ZP_00629446), *Rhodobacter sphaeroides* 2.4.1 (AAA26115), *Sinorhizobium meliloti* (CAC48591), *Aurantimonas* sp. SI85-9A1 (AAB41464), *Bradyrhizobium japonicum* USDA110 (BAC47850), *Chrysochromulina hirta* (P48692), *Cylindrotheca* sp. N1 (AAA84192), *Ectocarpus siliculosus* (P24313), *Emiliania huxleyi* (YP_277313), *Nitrosococcus oceani* ATCC19707 (ABA56859), *Nitrosospira multiformis* ATCC251966 (WP_011380043), *Odontella sinensis* (NP_043654), *Olisthodiscus luteus* (P14959), *Pleurochrysis carterae* (Q08051), *Porphyridium aerugineum* (Q09119), *Rhodopseudomonas palustris* BisA53 (WP_011662799), *Dechloromonas aromatica* RCB (AAZ48366), *Hydrogenovibrio marinus* (Q59462), *Lingulodinium polyedrum* (AAA98748), *Magnetospirillum magnetotacticum* (AAL76920), *Magnetospirillum magnetotacticum* AMB-1 (WP_011385076), *Polaromonas napthalenivorans* CJ2 (WP_011801368), *Rhodobacter capsulatus* (P50922), *Rhodoferax ferrireducens* T118 (WP_011463692), *Rhodopseudomonas palustris* BisA53 (WP_011664101), *Rhodospirillum rubrum* (CAA25080), *Symbiodinium* sp. (AAG37859), *Thiobacillus denitrificans* ATCC25259 (WP_011313150), *Methanocaldococcus jannaschii* (AAB99239), *Methanosarcina acetivorans* C2A (AAM07894), *Methanosarcina barkeri* fusaro (AAZ69876), *Methanosarcina mazei* Go1 (AAM30945), *Archaeoglobus fulgidus* DSM4304 (WP_010879134), *Hyperthermus butylicus* DSM5456 (WP_011821683), *Natronomonas pharaonis* DSM2160 (CAI49476), *Pyrococcus abyssi* GE5 (CAB50122), *Pyrococcus furiosus* DSM3638 (AAL81280), *Pyrococcus horikoshii* OT3 (BAA30036), *Thermococcus kodakaraensis* KOD1 (BAD864790, *Thermofilum pendens* Hrk5 (WP_011752890), *Acidiphilium cryptum* JF-5 (ZP_01146529), *Bordetella bronchiseptica* RB50 (CAE31534), *Burkholderia xenovorans* (*fungorum*) LB400 (ZP_00284840), *Chromohalobacter salexigens* DSM3043 (ZP_00471249), *Delftia acidovorans* SPH1 (ZP_01577127), *Fulvimarina pelagi* HTCC2506 (ZP_01438569), *Jannaschia* sp. CCS1 (WP_011456184), *Mesorhizobium loti* (BAB53192), *Polaromonas* sp. JS666 (ZP_00502320), Ocean Sampling expedition (EBH57905), Global Ocean Sampling expedition (EBM16636), Global Ocean Sampling expedition (EDE27295), Global Ocean Sampling expedition (EBO60441), Global Ocean Sampling expedition (EBK48460), *Alkalilimnicola ehrlichei* MLHE-1 (WP_011628912), *Archaeoglobus fulgidus* DSM4304 (WP_010879084), *Halorhodospira halophila* SL1 (WP_011813278), *Heliobacillus mobilis* (ABH04879), *Ostreococcus* *tauri* (Ot07g01830), *Ostreococcus* *tauri* (Ot08g02600), *Rhodopseudomonas* *palustris* BisA53 (WP_011665075), *Allochromatium* *vinosum* (BAB44150), *Chlorobium* *chlorochromatii* CaD3 (ABB28892), *Pelodictyon* *luteolum* DSM273 (ABB23300), *Pelodictyon* *phaeoclathratiforme* BU-1 (ZP_00590598), *Prosthecochloris* *aestuarii* DSM271 (ZP_00590874), *Rhodopseudomonas* *palustris* BisA53 (WP_011661875), *Bacillus* *anthracis* Ames (AAP27976), *Exiguobacterium* *sibiricum* 255-15 (ZP_00539172), *Geobacillus* *kaustophilus* HTA426 (BAD75238).

**Table S2**. Identification of genes associated with pathways indicated in **Figure 1**.

|  |  | **Gene annotation** | **Gene ID** |
| --- | --- | --- | --- |
| **Respiration** | Complex I | NADH-quinone oxidoreductase NuoABCDEFGHIJKLMN | GE002696, GE002697, GE002698, GE002699, GE002700, GE002701, GE002702, GE002703, GE002705, GE002706, GE002708, GE002709, GE002710, GE002711 |
|  | Complex Ⅱ | Succinate dehydrogenase SdhABCD | GE000608, GE000609, GE000610, GE000611 |
|  | Complex Ⅲ | Cytochrome *bc_1_* complex PetABC | GE001480, GE001481, GE001482 |
|  | Complex Ⅳ | Cytochrome *bd* ubiquinol oxidase CydAB | GE000280, GE000281 |
|  |  | *aa_3_*-type cytochrome *c* oxidase CtaCDEF | GE001298, GE001299, GE003138, GE003142, GE003143, GE003629 |
|  |  | *cbb_3_*-type cytochrome *c* oxidase CcoNOQP | GE003264, GE003265, GE003266, GE003267 |
|  | Complex Ⅴ | F_1_F_0_-type ATP synthase | GE000640, GE000641, GE000642, GE000643, GE000644 |
| **Phototrophy** | Photosynthetic reaction center | Photosynthetic reaction center cytochrome *c*, PufC | GE000917 |
|  |  | Photosynthetic reaction center M subunit PufM | GE000918 |
|  |  | Photosynthetic reaction center L subunit PufL | GE000919 |
|  |  | Photosynthetic reaction center subunit H PuhA | GE00061 |
|  | Light-harvesting complex LH1 | Light-harvesting protein B-880 alpha chain | GE000920 |
|  |  | Light-harvesting protein B-880 beta chain | GE000921 |
|  | Mono- or di-heme cytochrome *c* family protein | Cytochrome *c_2_* | GE001440 |
|  |  | Di-heme cytochrome *c*-type | GE003081 |
|  |  | Cytochrome *c_553_* | GE001333, GE001644, GE000260 |
|  |  | Cytochrome *c_556_* | GE001265, GE003082 |
| **Carbon metabolism** | Calvin-Benson-Bassham cycle | Form Ⅳ RuBisCo-like protein | GE001853, GE003255 |
|  |  | Phosphoglycerate kinase | GE003553 |
|  |  | Ribulose-5-phosphate 3-epimerase | GE002084 |
|  |  | Transketolase | GE003550 |
|  |  | Fructose-1,6-bisphosphatase | GE002740 |
|  |  | Glyceraldehyde 3-phosphate dehydrogenase | GE003551, GE000408 |
|  |  | Phosphoribulokinase | Not detected |
|  |  | Triose phosphate isomerase | GE002485 |
|  |  | Fructose-bisphosphate aldolase | GE003554 |
|  | Reductive tricarboxylic acid cycle | Citrate lyase | GE000532, GE000693, GE002145 |
|  |  | Fumarate reductase | GE002207 |
|  |  | α-Ketoglutarate synthase | Not detected |
|  |  | Aconitate hydratase | GE000580, GE000694 |
|  |  | Isocitrate dehydrogenase | GE002061 |
|  |  | Succinyl-CoA synthetase | GE000618, GE000619 |
|  |  | Fumarate hydratase | GE002269, GE003478 |
|  |  | Malate dehydrogenase | GE000617 |
|  | Glycolysis/gluconeogenesis | Phosphofructokinase | Not detected |
|  |  | Glucose-6-phosphatase | Not detected |
|  |  | Phosphoenolpyruvate carboxykinase | GE000459 |
|  |  | Enolase | GE001132, GE002479 |
|  |  | 2,3-Bisphosphoglycerate-independent phosphoglycerate mutase | GE000840, GE003152 |
|  |  | Phosphoglycerate kinase | GE003553 |
|  |  | Glyceraldehyde 3-phosphate dehydrogenase | GE000408, GE003551 |
|  |  | Fructose-bisphosphate aldolase | GE003554 |
|  |  | Fructose-1,6-bisphosphatase | GE002740 |
|  |  | Glucose-6-phosphate isomerase | GE001000 |
|  | Pentose phosphate pathway | Glucose-6-phosphate dehydrogenase | GE001162 |
|  |  | 6-Phosphogluconolactonase | GE001161 |
|  |  | 6-Phosphogluconate dehydrogenase | GE001801 |
|  |  | Ribose 5-phosphate isomerase | GE002113, GE003359 |
|  |  | Ribose-phosphate pyrophosphokinase | GE001491 |
|  |  | Glucose-6-phosphate isomerase | GE001000 |
|  |  | Fructose-1,6-bisphosphatase | GE002740 |
|  |  | Fructose-bisphosphate aldolase | GE003554 |
|  |  | Transketolase | GE003550 |
|  |  | Transaldolase | GE000637 |
|  |  | Ribulose-phosphate 3-epimerase | GE002084 |
|  | Inorganic carbon assimilation | Carbonic anhydrase | GE000512 |
| **Nitrogen metabolism** | | Urea transport system | GE000753, GE000754, GE000755, GE000756, GE000757 |
|  |  | Urease | GE001895, GE001896, GE001898 |
|  |  | Cyanate hydratase | GE003299 |
|  |  | Carbamoyl-phosphate synthase | GE001519, GE001523 |
|  |  | Nitrilase | GE000758, GE002865, GE003322, GE003733 |
|  |  | Ammonium transporter | GE000517 |
|  |  | Nitrogen regulatory protein pii | GE000518 |
|  |  | Nitronate monooxygenase | GE001010 |
|  |  | Nitrate/nitrite response regulator protein NarL | GE000344, GE001266 |
|  |  | Nitrate ABC transporter related protein | GE003296, GE003297, GE003298 |
|  |  | Nitrogen regulatory two-component system NtrB-NtrC | GE002297, GE002298 |
|  |  | Nitrogen regulatory two-component system NtrY-NtrX | GE002299, GE002300 |


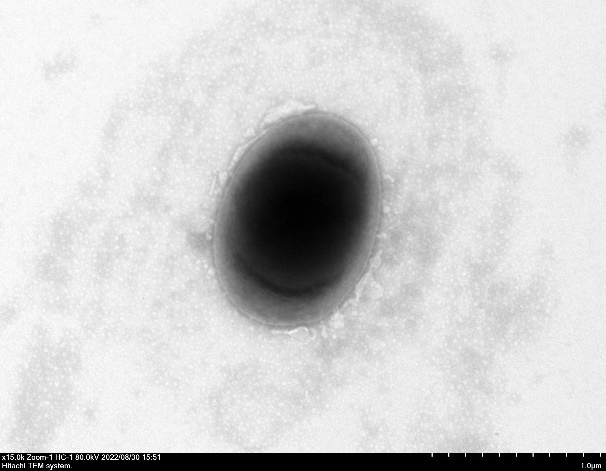


**Figure S1** Transmission electron microscope image of strain MAB10.

**
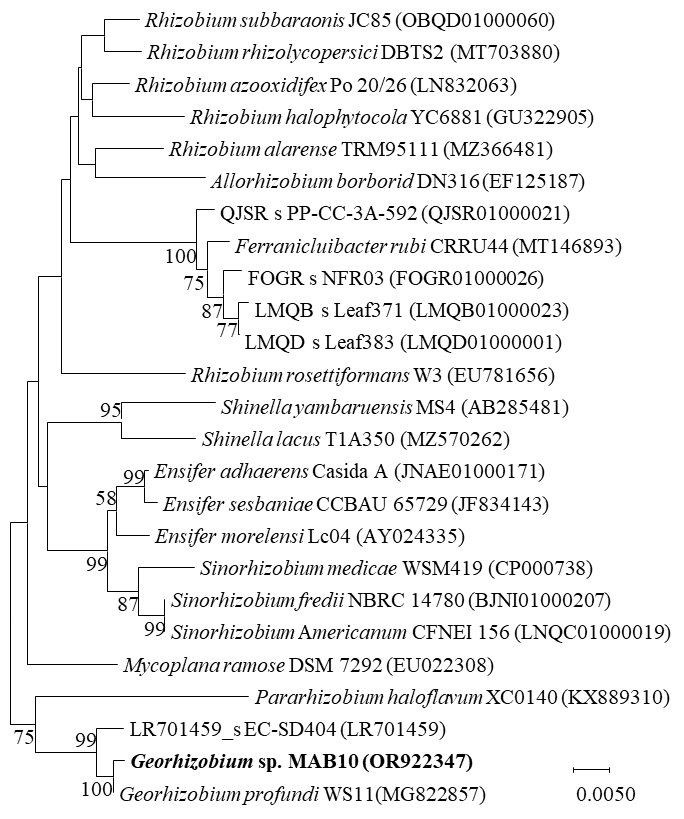
**

**Figure S2** Neighbor-joining phylogenetic tree of full-length 16S rRNA genes of strains MAB10, WS11, and selected alpha-proteobacteria isolates. Bootstrap values greater than 50% are displayed at nodes (1000 replicates). The NCBI GenBank accession numbers of 16S rRNA gene sequences are shown in parentheses.


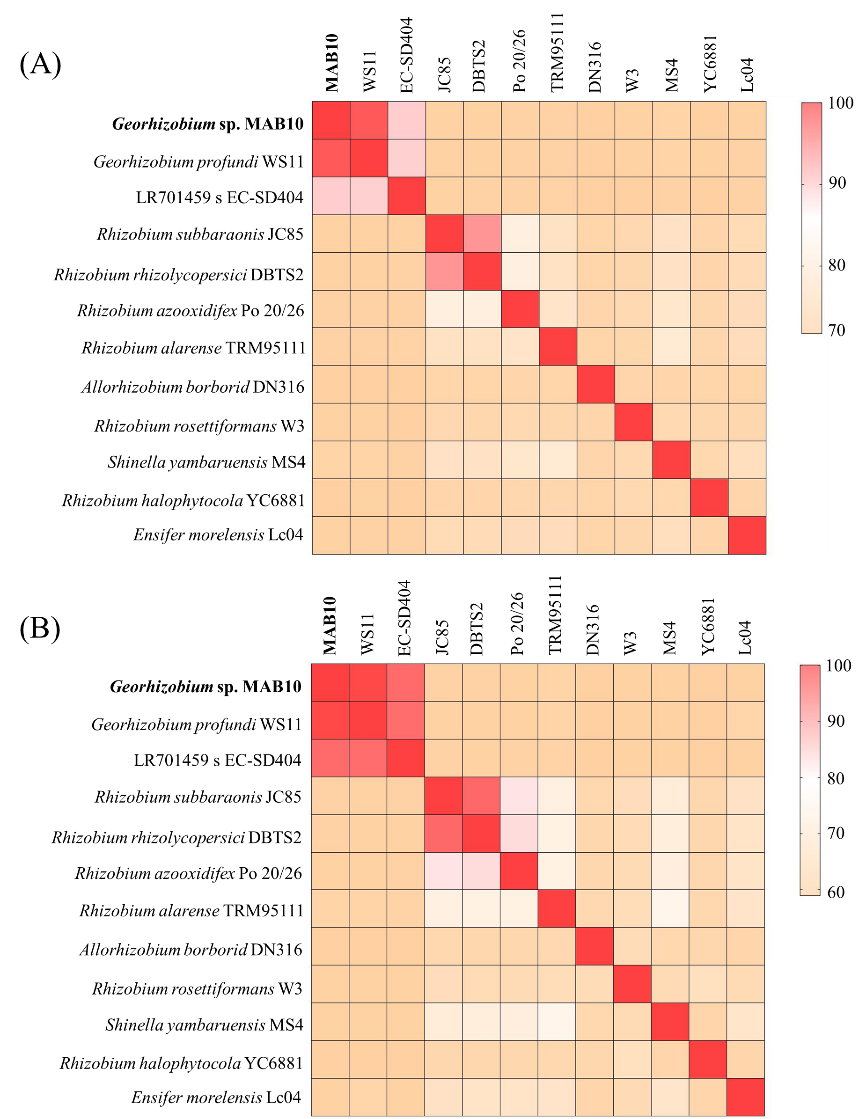


**Figure S3** Heatmaps based on ANI (A) and average AAI (B) between strains MAB10 as well as selected alpha-proteobacteria isolates.


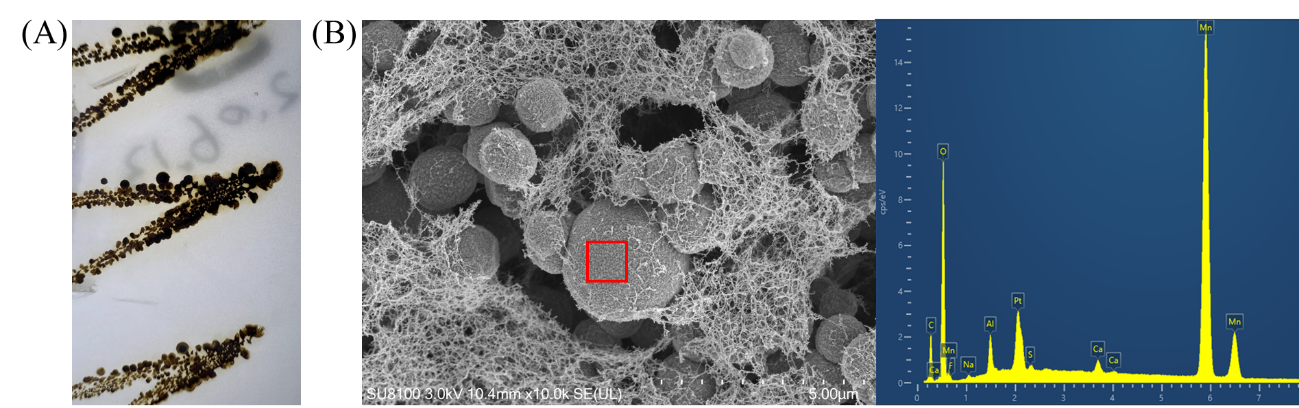


**Figure S4** Scanning electron microscope-energy dispersive system image of Mn-oxides formed by strain MAB10 in agarose-solidified basal medium supplemented with MnCO_3_.


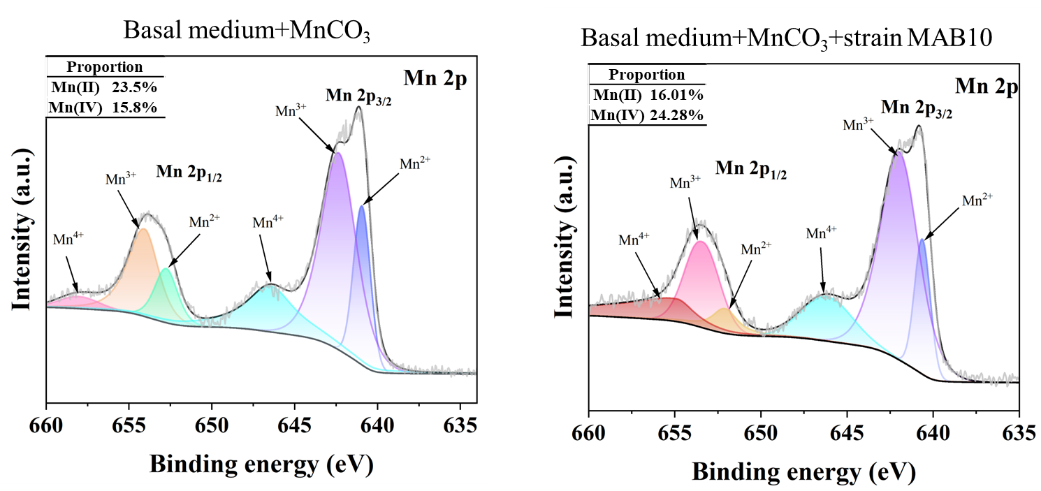


**Figure S5** Mn (2p) spectrograms of Mn-precipitates with or without inoculation of strain MAB10.


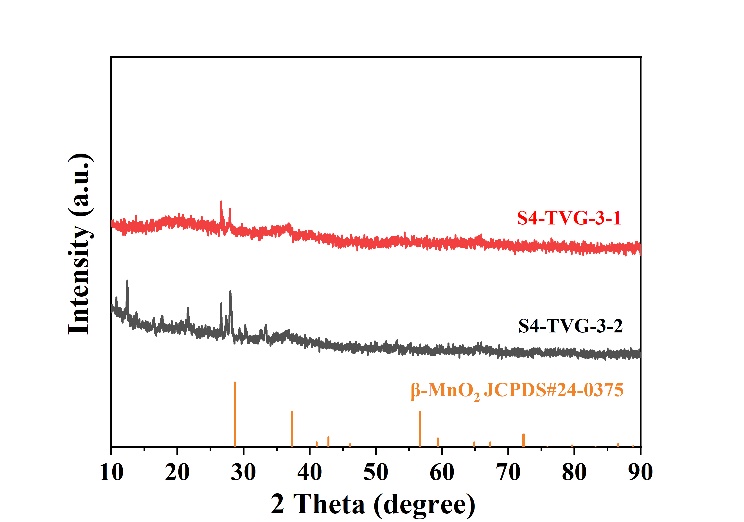


**Figure S6** XRD analysis result of the deep-sea ferromanganese nodule sample (S4-TVG-3) from which strain MAB10 isolated.

**
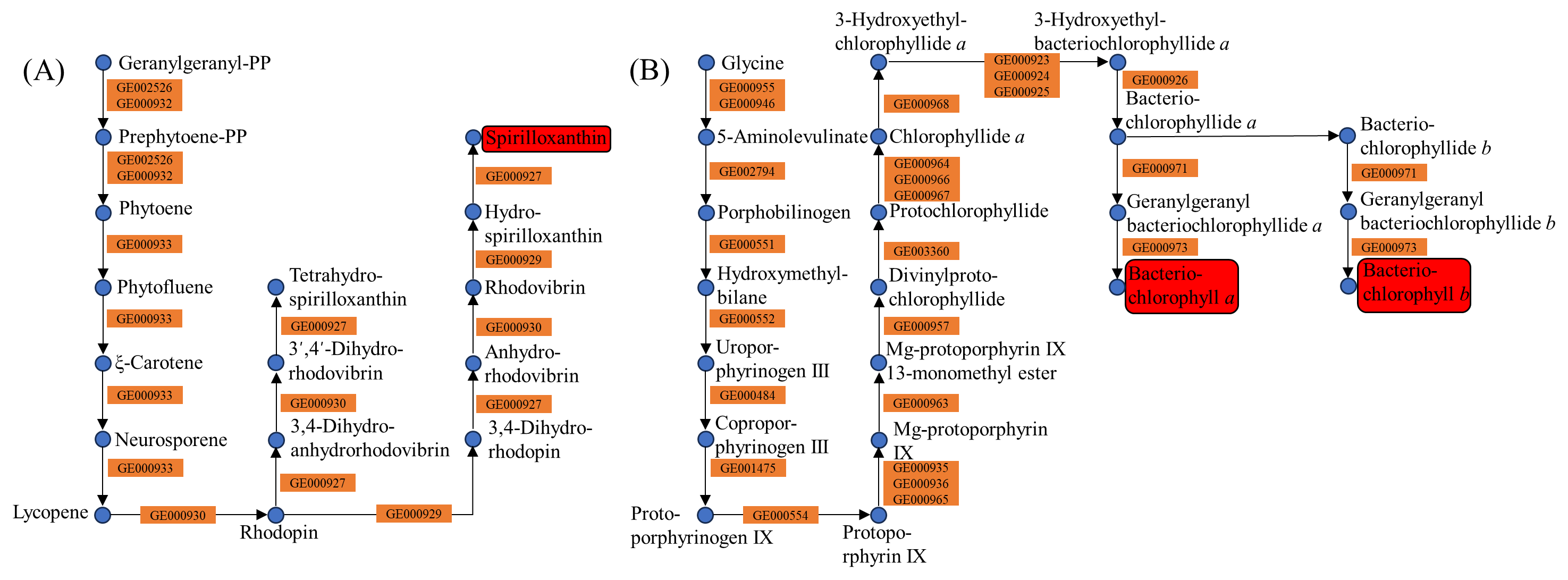
**

**Figure S7** Complete biosynthesis of bacteriochlorophyll *a* and *b* (A) and major carotenoid spirilloxanthin (B) pathways in strain MAB10.

**
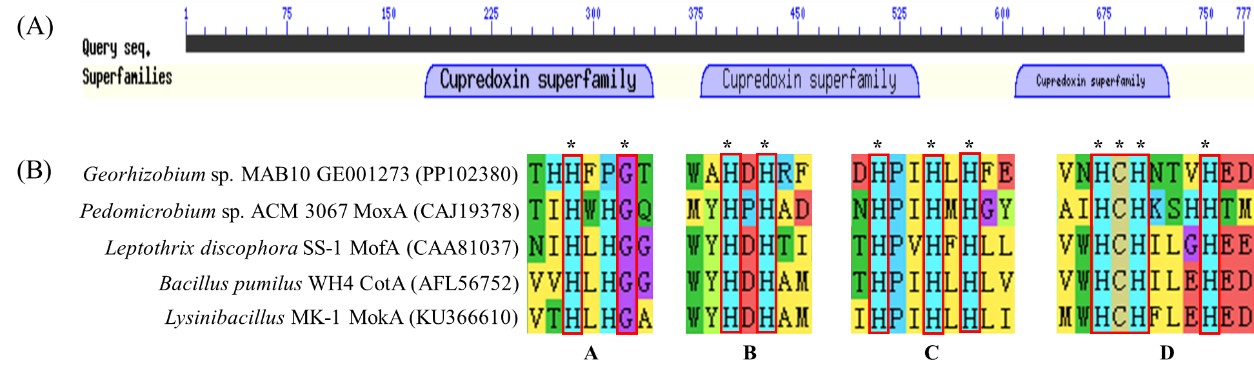
**

**Figure S8** Amino acid sequence analysis of GE001273. (A), BLAST result of GE001273. (B), Amino acid sequence alignment of the four conserved Cu-binding sites of MCOs from diverse bacterial strains. Regions A, B, C, and D represent Cu-binding regions. The Cu-binding residues are designated in red boxes.


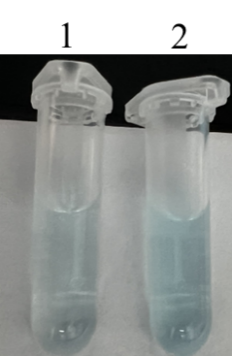


**Figure S9** LBB test for Mn-oxides formation catalyzed by GE001273. Tube 1, 10 mM HEPES buffer (pH 7.5) plus MnCl_2_ and CuCl_2_ reacting with LBB; tube 2, aliquots of tube 1 plus purified GE001273.
